# Stochastic Resonance with Parameter Estimation for Enhancing Unknown Compound Fault Detection of Bearings

**DOI:** 10.3390/s23083860

**Published:** 2023-04-10

**Authors:** Min Xu, Chao Zheng, Kelei Sun, Li Xu, Zijian Qiao, Zhihui Lai

**Affiliations:** 1 Ningbo Cigarette Factory, China Tobacco Zhejiang Industry Co., Ltd., Ningbo 315040, China; 2 School of Mechanical Engineering and Mechanics, Ningbo University, Ningbo 315211, China; 3 Yangjiang Offshore Wind Power Laboratory, Yangjiang 529500, China; 4 State Key Laboratory of Performance Monitoring and Protecting of Rail Transit Infrastructure, East China Jiaotong University, Nanchang 330013, China; 5 Zhejiang Provincial Key Laboratory of Part Rolling Technology, Ningbo 315211, China; 6 Shenzhen Key Laboratory of High Performance Nontraditional Manufacturing, College of Mechatronics and Control Engineering, Shenzhen University, Shenzhen 518060, China

**Keywords:** stochastic resonance, weak fault detection, mechanical fault diagnosis

## Abstract

Although stochastic resonance (SR) has been widely used to enhance weak fault signatures in machinery and has obtained remarkable achievements in engineering application, the parameter optimization of the existing SR-based methods requires the quantification indicators dependent on prior knowledge of the defects to be detected; for example, the widely used signal-to-noise ratio easily results in a false SR and decreases the detection performance of SR further. These indicators dependent on prior knowledge would not be suitable for real-world fault diagnosis of machinery where their structure parameters are unknown or are not able to be obtained. Therefore, it is necessary for us to design a type of SR method with parameter estimation, and such a method can estimate these parameters of SR adaptively by virtue of the signals to be processed or detected in place of the prior knowledge of the machinery. In this method, the triggered SR condition in second-order nonlinear systems and the synergic relationship among weak periodic signals, background noise and nonlinear systems can be considered to decide parameter estimation for enhancing unknown weak fault characteristics of machinery. Bearing fault experiments were performed to demonstrate the feasibility of the proposed method. The experimental results indicate that the proposed method is able to enhance weak fault characteristics and diagnose weak compound faults of bearings at an early stage without prior knowledge and any quantification indicators, and it presents the same detection performance as the SR methods based on prior knowledge. Furthermore, the proposed method is more simple and less time-consuming than other SR methods based on prior knowledge where a large number of parameters need to be optimized. Moreover, the proposed method is superior to the fast kurtogram method for early fault detection of bearings.

## 1. Introduction

Rolling bearings are key components of rotating machinery and can provide reliable and stable support. However, during the operating process, rolling bearings are inevitable; their defects include wear, cracking and pitting due to insufficient lubrication, contact fatigue, etc. Therefore, it is very important for us to achieve early fault detection and fault diagnosis of bearings to avoid a serious accident. Up until now, there were two well-known categories of bearing fault diagnosis, including model updating [1,2] and direct feature extraction and analysis using machine learning [3,4] or signal processing [5,6]. In addition, some scholars focus on the prediction of remaining useful life [7,8].

Among them, because direct feature extraction and analysis based on signal processing does not require building complex mathematical models and complete fault samples, they have attracted sustained attention in fault detection. However, most signal processing methods attempt to cancel the noise embedded in signals to detect weak fault features, while stochastic resonance is able to harvest the energy of noise to enhance weak useful signals and therefore has been widely used in weak fault detection and fault diagnosis of machinery. In 2019, Lu et al. [9] and Qiao et al. [10] reviewed these articles on SR-based fault diagnosis of machinery and pointed out the potential development directions, respectively. Two review articles are inspiring researchers to explore the potential of SR as well as to develop advanced research in this field.

Up until now, there were many scholars who investigated the application of SR in mechanical fault diagnosis. Among them, some paid attention to designing novel indicators to quantify SR. For example, López et al. [11] proposed hidden Markov models and a Box–Cox sparse measures-based SR method for bearing fault diagnosis in which the designed indicator does not depend on prior knowledge of the fault signature to be detected, but the parameter selection of hidden Markov models and Box–Cox sparse measures would complicate the proposed method. Lin et al. [12] designed a novel indicator to quantify the SR for bearing fault diagnosis, where the cross-correlation coefficient, impulse index and signal-to-noise ratio (SNR) are fused. Li et al. [13] presented a multi-parameter constrained potential underdamped SR method and applied it to weak fault diagnosis, where SNR is used to evaluate SR. Lai et al. [14] defined SNR_input_ and SNR_output_ as the signal-to-noise ratios at SR system input and output to quantify SR for mechanical fault diagnosis. Zhang et al. [15] used the opposite of SNR as a fitness function of salp swarm algorithms to optimize the parameters of the proposed SR method. Wang et al. [16] applied order tracking to address the time-varying signals and then designed a tristable SR method to enhance weak fault characteristics with the fitness function of SNR. Zhou et al. [17] used generative adversarial networks to fuse 10 statistical parameters as the fitness function for optimizing the proposed SR method. Shi et al. [18] proposed a novel adaptive multi-parameter unsaturation bistable SR method in which the output SNR is selected as the objective function. Qiao et al. [19] proposed a second-order SR method enhanced by a fractional-order derivative for mechanical fault detection where SNR is used to optimize its parameters, even in coupled neurons [20] or the proposed nonlinear resonant decomposition [21]. Li et al. [22] presented a frequency-shift multiscale noise tuning SR method for fault diagnosis of generator bearings in wind turbines and even presented the coupled bistable SR method [23] in which the parameters of the proposed method are optimized through modified SNR and genetic algorithms. Fu et al. [24] studied the SR in Duffing oscillators and proposed a moment-method-based bearing fault diagnosis algorithm where the output SNR is seen as the objective function of optimization algorithms. Xu et al. [25] studied the SR behaviors in a high-order-degradation bistable system, and the ratio of the amplitudes around the target orders to those of the interference orders was calculated as the objective function of optimization algorithms. In summary, most of the proposed SR methods’ objective functions depend on prior knowledge of the fault characteristics to be detected, which would result in false SR and weaken the detection performance of SR further. It is improper to enhance and detect unknown weak fault characteristics, especially for real-world equipment. In addition, a large number of tuning parameters in the existing SR methods need to be optimized by using artificial intelligence, which is very time-consuming and complex and is not suitable to engineering application.

Triggering SR has clear mathematical conditions, but among these variables in the conditions, the intensity of noise is indefinite. If we could estimate the intensity of noise in a signal, a parameter-matching equation could be built. Motivated by such an idea, this paper will attempt to design a parameter-matched second-order SR method for enhancing unknown weak fault characteristics of bearings due to second-order SR that has a bandpass filtering property. The remainder of this paper is organized as follows. Section 2 gives clear mathematical conditions of parameter-matched second-order SR and then proposes the corresponding SR method for bearing fault enhancement. In Section 3, a bearing fault experiment was performed to demonstrate the effectiveness of the proposed method, and a comparison is made. Finally, conclusions are drawn in Section 4.

## 2. A Parameter-Matched Second-Order SR Method

A second-order SR can be described as below [26],

(1) $$\frac{d^{2}x}{dt^{2}}+\gamma \frac{dx}{dt}=-\frac{dV\left(x\right)}{dx}+s\left(t\right)+n\left(t\right)$$

where γ is the damping factor and $0<\gamma \leq 2\sqrt{2a}$ [27], $s\left(t\right)$ is a periodic signal to be detected and its expression can be written as $A\cos\left(2\pi f_{0}t+\varphi \right)$ with the amplitude A, driving frequency $f_{0}$ and initial phase φ. $n\left(t\right)$ is noise with $\langle n\left(t\right),n\left(t+\tau \right)\rangle =2D\delta \left(t\right)$ and D as the noise intensity. $V\left(x\right)$ is the bistable potential as below,

(2) $$V\left(x\right)=-\frac{a}{2}x^{2}+\frac{b}{4}x^{4},a,b>0$$

where two stable states and one unstable state are located at $\pm x_{m}=\pm \sqrt{a/b}$ and $x_{0}=0$, respectively. Moreover, the barrier height is $∆V=\frac{a^{2}}{4b}$. According to two-state theory, the matched conditions for triggering the SR induced by a periodic signal is [28]

(3) $$r_{K}=2f_{0}$$

where $r_{K}$ is the Kramers’ rate given by [29]

(4) $$r_{K}=\frac{\omega_{b}\omega_{0}}{2\pi \gamma }\exp\left(-\frac{∆V}{D}\right)$$

in which $\omega_{b}=\sqrt{2a}$ and $\omega_{0}=\sqrt{a}$.

For convenience, we rewrite the matched condition in Equation (3) as [30]

(5) $$F\left(a,b,D,\gamma ,f_{0}\right)=\frac{a}{2\sqrt{2}\pi \gamma f_{0}}\exp\left(-\frac{a^{2}}{4bD}\right)$$

It can be noticed from Equation (5) that SR can be induced when F=1. In general, the input and output SNR in Equation (1) for a periodic signal plus additive noise can be written as [31]

(6) $$SNR_{\mathrm{input}}=\frac{A^{2}}{4D}$$

(7) $$SNR_{\mathrm{output}}\approx \frac{aA^{2}\sqrt{2a}}{4bD^{2}}\exp\left(-\frac{a^{2}}{4bD}\right)$$

Then, the SNR gain (Signal-to-noise ratio gain, SNRG) can be calculated as

(8) $$SNRG=\frac{SNR_{\mathrm{output}}}{SNR_{\mathrm{input}}}\approx \frac{a\sqrt{2a}}{bD}\exp\left(-\frac{a^{2}}{4bD}\right)$$

It can be seen from Equation (8) that the SNRG would be controlled by bistable potential parameters and the noise intensity. Thus, the objective function of the signal to be detected is given as the following optimization problem:

(9) $$D_{opt}=\mathrm{argmax}\;SNRG$$

By solving Equation (8) with respect to D, we can obtain the optimal condition as below:

(10) $$D_{opt}=\frac{a^{2}}{4b}=∆V$$

Therefore, the optimal parameter-matched condition for weak fault enhancement can be given as below:

(11) $$\left\{\begin{array}{c} F=1\\ D=\frac{a^{2}}{4b} \end{array}\right.$$

However, Equation (1) is suitable to process weak signals subject to small-parameter limitation. In the real world, the mechanical signals are generally large parameter. For solving this issue, a normalized scale transformation is performed to Equation (1). Let $z=x\sqrt{b/a}$ and τ=at, Equation (1) could be rewritten as

(12) $$\frac{d^{2}z}{d\tau^{2}}+\gamma_{h}\frac{dz}{d\tau }=a_{h}z-b_{h}z^{3}+A_{h}\cos\left(2\pi f_{0h}\tau +\varphi \right)+D_{h}\xi \left(\frac{\tau }{a}\right)$$

where $\gamma_{h}=\gamma /a$, $a_{h}=b_{h}=1/a$, $f_{0h}=f_{0}/a$, $A_{h}=\sqrt{b/a^{5}}A$, and $D_{h}=b/a^{5}D$. By comparison, it is found that large-parameter signals can be transferred into small-parameter ones in which the amplitude is reduced by the time of $\sqrt{b/a^{5}}$ and the frequency is reduced by the time of 1/a. By substituting the above parameters in Equation (12) into Equation (11), the parameter-matched conditions for triggering SR for weak large-parameter signal detection can be achieved as below:

(13) $$\left\{\begin{array}{c} F\left(a_{h},\;b_{h},\;D_{h},\;f_{0h}\right)=\frac{a_{h}}{2\sqrt{2}\pi \gamma_{h}f_{0h}}\exp\left(-\frac{a_{h}^{2}/4b_{h}}{D_{h}}\right)=1\\ D_{h}=a_{h}^{2}/4b_{h} \end{array}\right.$$

which is calculated further as

(14) $$\left\{\begin{array}{c} a=2\sqrt{2}\pi f_{0}\gamma e\\ b=a^{4}/4D \end{array}\right.$$

It can be seen from Equation (14) that the damping factor γ can be adjusted to enhance the weak fault characteristics of bearings. According to the condition $0<\gamma_{h}\leq 2\sqrt{2a_{h}}$, we can obtain the limitation.

(15) $$0<\gamma <16\sqrt{2}\pi f_{0}e$$

**Figure 1:**
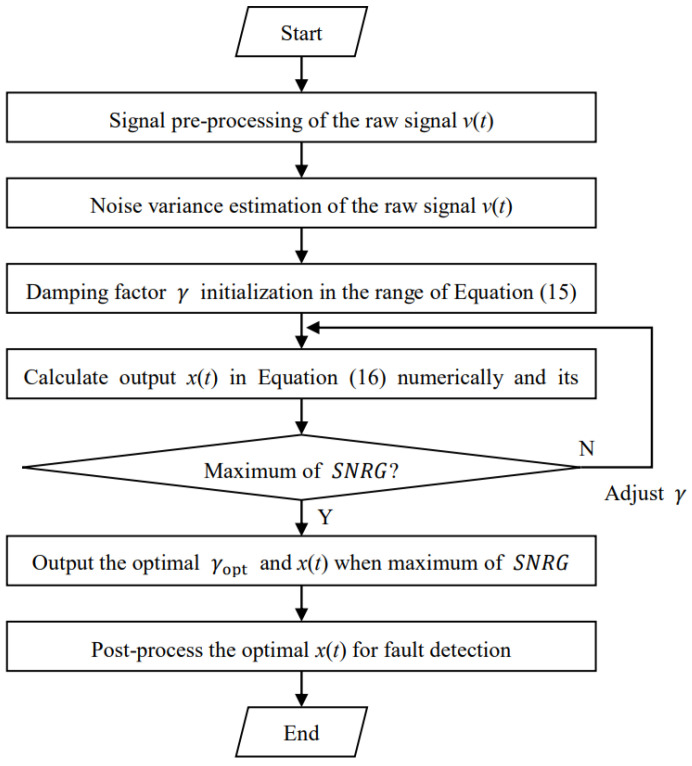
The flowchart of the proposed method.

Therefore, the parameter-matched conditions can be given by using Equations (14) and (15) mathematically for weak large-parameter signal detection. According to the above parameter-matched conditions, we can design an SR method with parameter estimation to enhance the weak large-parameter fault signature of bearings. The proposed method is shown in Figure 1, and its detailed steps are given as below.

1. Signal pre-processing. In general, the bearing vibration signal *v*(*t*) is a large-parameter signal where the bearing fault characteristics are modulated by natural vibration from the machinery itself. Hence, some signal demodulations are performed to the raw signals. Here, Hilbert envelope demodulation is used to pre-process the raw vibration signal of the tested bearing, and the corresponding envelope signal is denoted as $\tilde{v}\left(t\right)$.
2. Noise intensity estimation. In Equation (14), an important parameter D, namely, noise intensity, needs to be estimated from the envelope signal $\tilde{v}\left(t\right)$ of the raw signal of the tested bearing. Here, we use the principle of maximum likelihood estimation (MLE) [32] to achieve this, and we can obtain $D=MLE\left(\tilde{v}\left(t\right)\right)$. The MLE algorithm can be downloaded by using the following website: http://www.biomecardio.com/matlab/evar_doc.html (accessed on 5 December 2022).
3. Damping factor initialization. The damping factor needs to be tuned to obtain the optimal detected result. Here, according to its range in Equation (15), we initialize the damping factor γ.
4. Output signal calculation and evaluation. By substituting $a_{\mathrm{match}}=2\sqrt{2}\pi f_{0}\gamma e$, $b_{\mathrm{match}}=a^{4}/4D$ and the corresponding damping factor into Equation (16), we can obtain the output signal $x\left(t\right)$. Then, we can calculate the corresponding SNRG as the objective function $\gamma_{\mathrm{opt}}=\mathrm{argmax}\;SNRG$ for optimizing the damping factor. Finally, the optimal $\gamma_{\mathrm{opt}}$ is substituted into Equation (17) to solve the optimal $x\left(t\right)$.
  (16) $$\frac{d^{2}x}{dt^{2}}+\gamma \frac{dx}{dt}=a_{\mathrm{match}}x-b_{\mathrm{match}}x^{3}+\tilde{v}\left(t\right)$$
  (17) $$\frac{d^{2}x}{dt^{2}}+\gamma_{\mathrm{opt}}\frac{dx}{dt}=a_{\mathrm{match}}x-b_{\mathrm{match}}x^{3}+\tilde{v}\left(t\right)$$
5. Signal post-processing. Output the optimal $x\left(t\right)$ corresponding to the maximum of SNRG as the detected signal. Here, the frequency spectral analysis is used to process the optimal $x\left(t\right)$ for observing the spectral peaks at the fault characteristic frequencies of bearings.

## 3. Bearing Fault Experimental Verification

**Figure 2:**
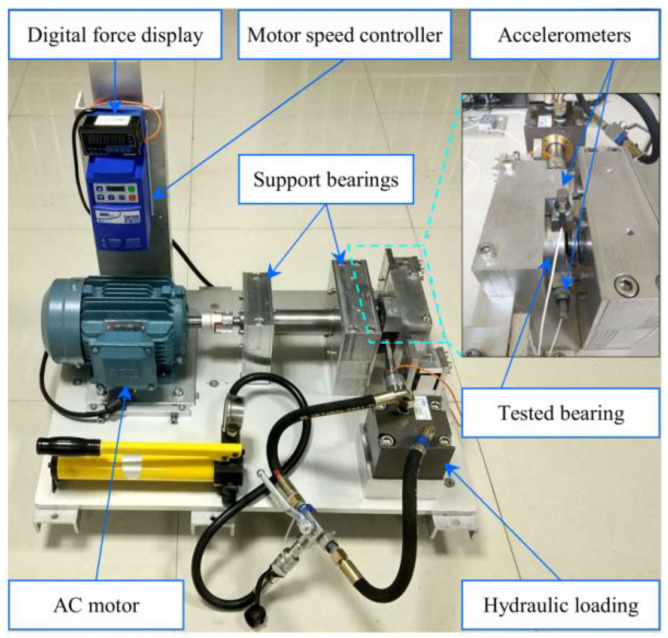
Bearing fault experimental setup.

The bearing fault experimental setup is shown in Figure 2 where a bearing accelerated degradation test was performed throughout the whole operating life of the bearing, and the corresponding vibration signals were acquired by using two sensors [33]. The two sensors were placed on the housing of the tested bearings and positioned at 90 degrees to each other, i.e., one was placed on the vertical axis and the other one was placed on the horizontal axis. The tested bearing’s parameters are given in Table 1. The sampling frequency was 25.6 kHz and 32,768 samples (i.e., 1.28 s) were recorded every 1 min. In addition, the rotating speed was 2100 rpm, and the radial force was 12 kN.

**Figure 3:**
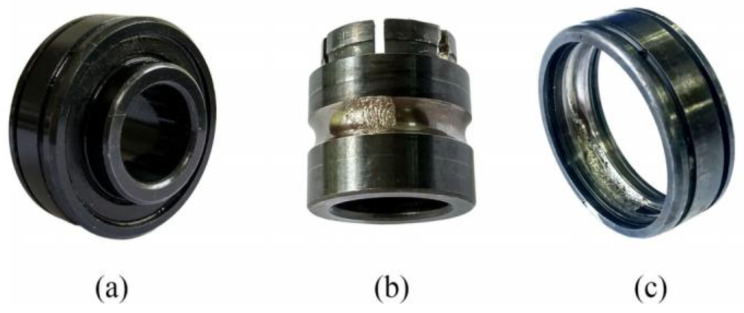
Photos of tested bearings: (**a**) Normal bearing, (**b**) Inner race wear and (**c**) Outer race wear.

**Figure 4:**
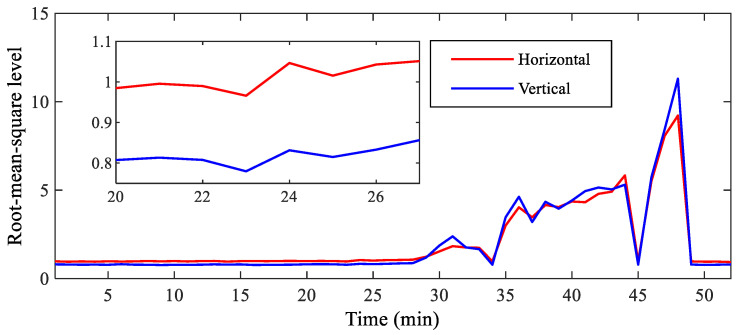
Horizontal and vertical RMS indicators of the tested bearing throughout its whole operating life.

**Figure 5:**
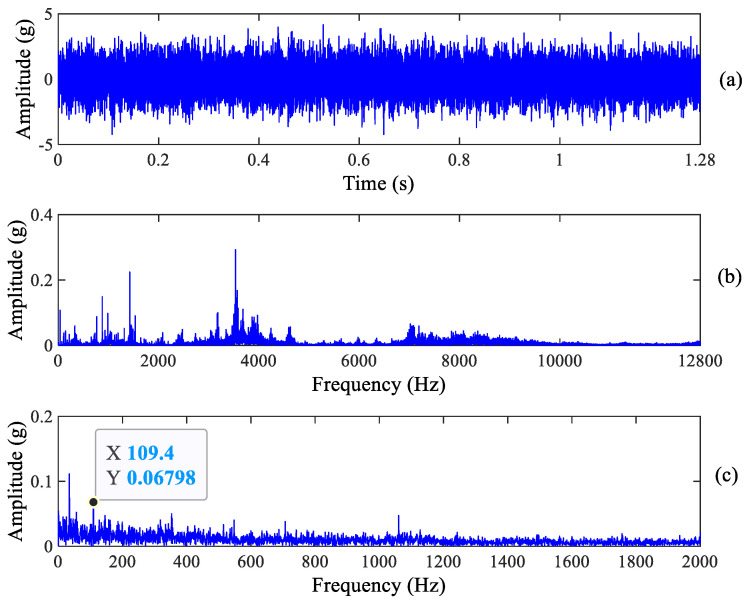
Horizontal raw vibration signal and its spectrum. (**a**) Raw signal, (**b**) Frequency spectrum and (**c**) Zoomed envelope spectrum.

**Figure 6:**
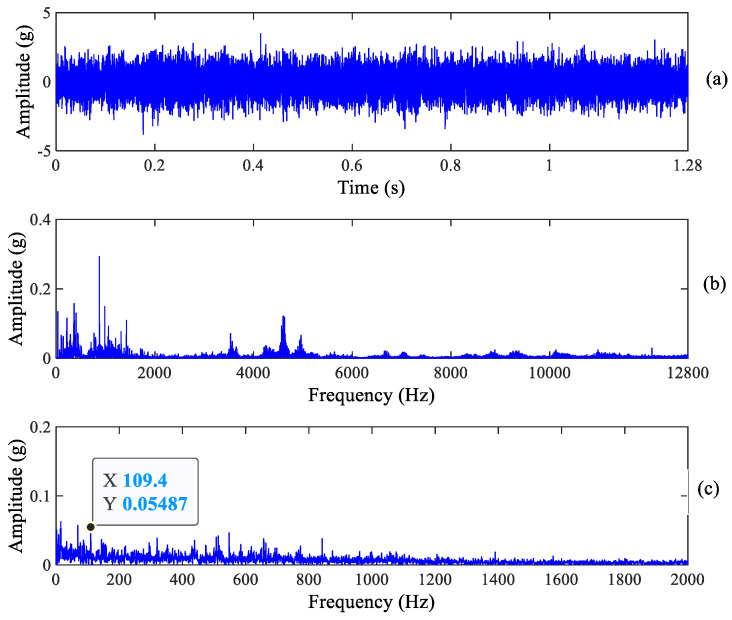
Vertical raw vibration signal and its spectrum: (**a**) Raw signal, (**b**) Frequency spectrum and (**c**) Zoomed envelope spectrum.

In the process of the bearing accelerated degradation test, inner race and outer race wear occurred in the tested bearing, as shown in Figure 3. Figure 4 depicts the horizontal and vertical root mean square (RMS) indicators of the tested bearing throughout its whole operating life. It can be seen from Figure 3 that horizontal and vertical RMS indicators were kept unchanged initially and then rose rapidly after 30 min, suggesting that early wear may have occurred in the tested bearing. According to the horizontal and vertical RMS indicators, we can deduce that early compound wear may happen in the inner race and outer race of the tested bearing at the 24th min. Therefore, the horizontal and vertical raw vibration signals and their spectrum are shown in Figure 5 and Figure 6, respectively. In the figures, we mark the inner race, outer race, roller and cage fault characteristic frequencies in the envelope spectrum by using different colors. According to the tested bearing’s parameters, we can calculate the theoretical inner race, outer race, roller and cage fault characteristic frequencies as 172.09, 107.91, 72.33 and 13.49 Hz, respectively, by using the following equations:

(18) $$f_{inner}=\frac{N}{2}f_{r}\left(1+\frac{d}{D}\cos\theta \right)$$

(19) $$f_{outer}=\frac{N}{2}f_{r}\left(1-\frac{d}{D}\cos\theta \right)$$

(20) $$f_{cage}=\frac{1}{2}f_{r}\left(1-\frac{d}{D}\cos\theta \right)$$

(21) $$f_{roller}=\frac{D}{d}f_{r}\left(1-{\left(\frac{d}{D}\cos\theta \right)}^{2}\right)$$

where N is the number of balls, $f_{r}$ is the rotating frequency, d is the ball diameter, D is the mean diameter and θ is the contact angle.

Compared with the information in the envelope spectrum in Figure 5 and Figure 6, it is found that there is a clear spectrum peak at 109.4 Hz which is close to the theoretical value of the outer race fault characteristic frequency 107.91 Hz, but the spectrum peaks of its harmonics cannot be recognized. Obviously, the above signature demonstrates that early outer race wear occurs, which is consistent with the real experimental results. In addition, we can see from Figure 5 that there is a clear spectrum peak at 68.75 Hz, which is not a roller fault characteristic frequency. Other information cannot be observed from Figure 5 and Figure 6. As a result, we can deduce that early outer race wear occurs in the tested bearing from the raw signal and its spectrum.

**Figure 7:**
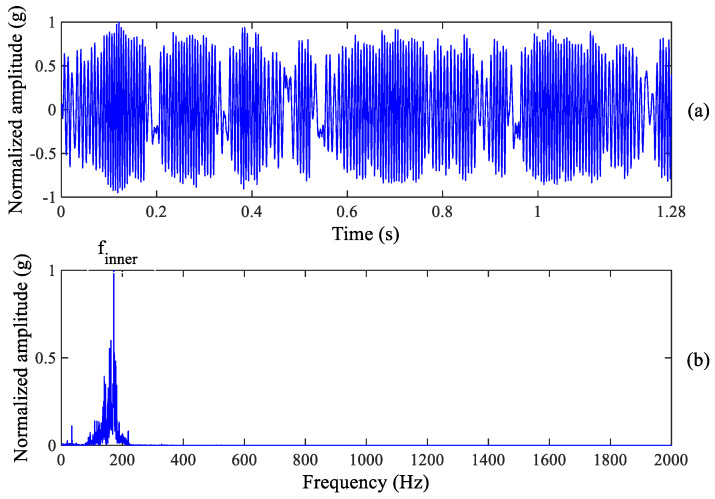
Inner race wear diagnosis result: (**a**) Enhanced signal (**b**) Zoomed frequency spectrum.

**Figure 8:**
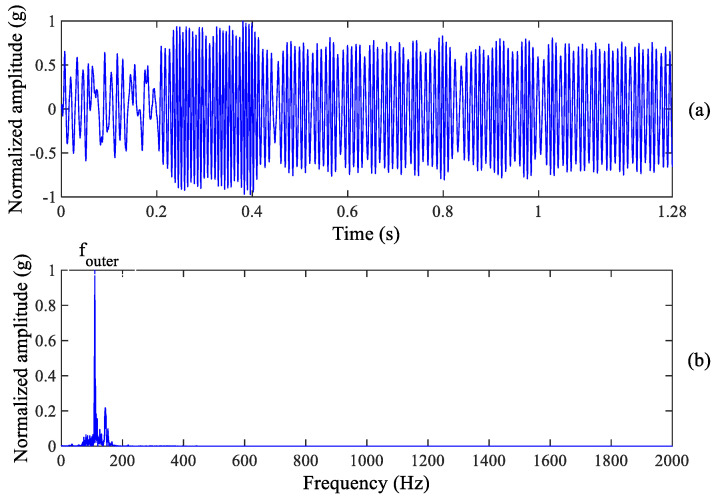
Outer race wear diagnosis result: (**a**) Enhanced signal (**b**) Zoomed frequency spectrum.

Although we can observe the weak spectrum peak at the outer race fault characteristic frequency, it cannot recognize the fault information excited by inner race wear. Therefore, the proposed method is used to process the raw vibration signal of the tested bearing. Inner race and outer race fault information can be enhanced by using the proposed method, as shown in Figure 7 and Figure 8. It is found from Figure 7b that there is a clear spectrum peak at 171.9 Hz which is close to the theoretical value of the inner race fault characteristic frequency 172.09 Hz, suggesting that early inner race wear happens in the tested bearing. In fact, an inner race wear has been formed as shown in Figure 3c. Meanwhile, we can also observe the clear spectrum peak at 109.4 Hz from Figure 8b which is close to the theoretical value of the outer race fault characteristic frequency 107.91 Hz, indicating that early outer race wear happens. The period of enhanced signal in Figure 7a and Figure 8a is equal to an inner race or outer race fault period, respectively.

**Figure 9:**
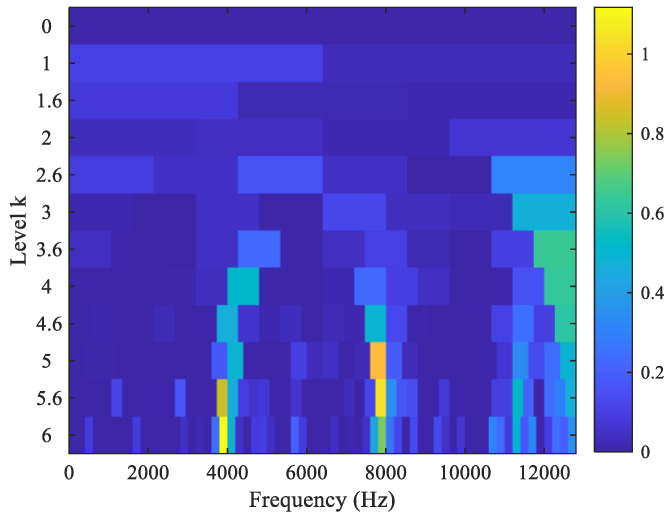
The kurtogram using the fast kurtogram method.

**Figure 10:**
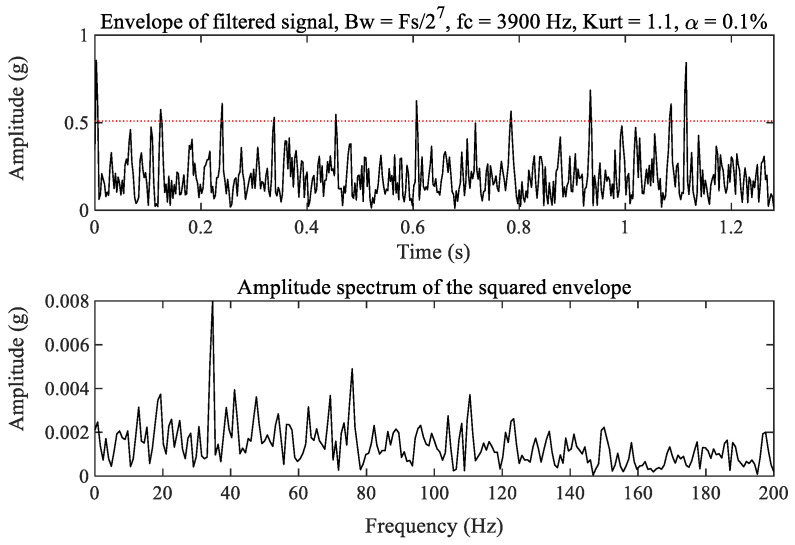
The filtered signal with the optimal carrier frequency 3900 Hz.

**Figure 11:**
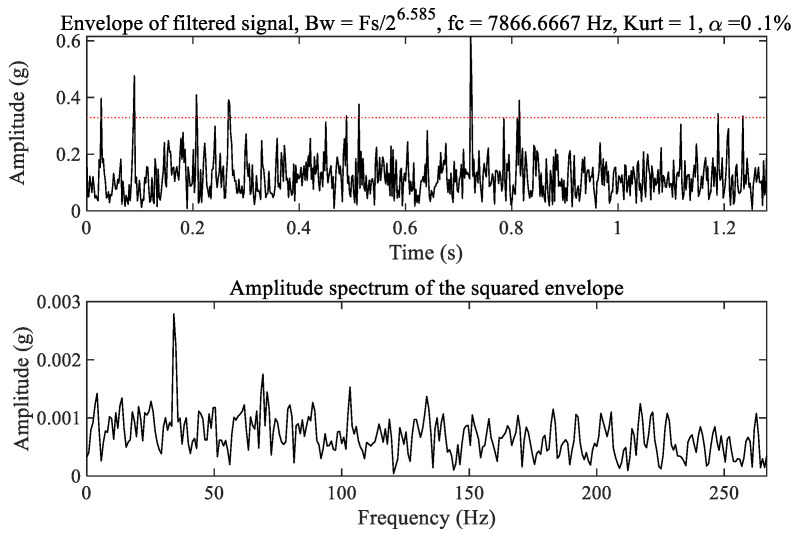
The filtered signal with the optimal carrier frequency 7866 Hz.

For comparison, the fast kurtogram [34,35], as a widely used method in mechanical fault diagnosis, is applied to process the raw vibration signal of the tested bearing. The kurtogram as shown in Figure 9 selects the optimal filtering frequency band as the optimal carrier frequency 3900 Hz with bandwidth 200 Hz to filter out the weak fault signature excited by the outer race and inner race wear in the tested bearing. The corresponding filtered result is shown in Figure 10. It can be seen from the amplitude spectrum of the filtered signal squared envelope in Figure 10 that there are clear spectrum peaks at 34.68 Hz and even at 75.81 Hz. Obviously, these are not fault signatures excited by inner race and outer race wear of the tested bearing. Therefore, we deduce that the optimal filtering frequency band may be the optimal carrier frequency 7866 Hz, as shown in Figure 9. The corresponding filtered signal is depicted in Figure 11. We cannot also observe clear spectrum peaks at the inner race and outer race fault characteristic frequencies from the amplitude spectrum of the filtered signal squared envelope in Figure 11. As a result, the fast kurtogram fails to detect the weak fault characteristics of the tested bearing in this experiment. The comparison demonstrates the feasibility and superiority of the proposed method.

## 4. Conclusions

Stochastic resonances (SR) have been widely used in mechanical fault diagnosis, but most of them require designing the quantification indicators by virtue of prior knowledge of the fault signature to be detected, resulting in false SR and further impaired detection performance of SR. Therefore, it is very necessary for us to study the parameter-matched SR method based on the mathematical condition of triggering SR. For this purpose, we propose an SR method with parameter estimation for bearing fault diagnosis. Compared with other SR methods, the tuning parameters of the proposed method can be estimated by virtue of the raw signal of bearings and do not depend on prior knowledge of the defects to be detected. Moreover, the proposed method considers the conditions of triggering SR to avoid false SR and improve the detection performance of SR further. Such an advantage makes the proposed method more suitable in engineering application. In addition, the proposed method has a tuning parameter and a damping factor with clear mathematical limitation, making it more simple and less time-consuming than other SR methods with a large amount of parameter optimization. A bearing fault experiment was performed to verify this. The experimental results indicate that the proposed method can enhance the weak compound fault diagnosis of bearings at an early stage and is superior to the widely used fast kurtogram method.

## Figures and Tables

**Table 1:** The tested bearing’s parameters.

Parameters	Values	Parameters	Values
Outer race diameter	39.80 mm	Inner race diameter	29.30 mm
Mean diameter	34.55 mm	Ball diameter	7.92 mm
Number of balls	8	Contact angle	0°

## Data Availability

The datasets generated during and/or analyzed during the current study are available from the corresponding author on reasonable request.
